# *Delirium* and sleep quality in the intensive care unit: the role of melatonin

**DOI:** 10.62675/2965-2774.20240083-en

**Published:** 2024-04-26

**Authors:** Pedro Henrique Rigotti Soares, Rodrigo Bernardo Serafim

**Affiliations:** 1 Hospital Nossa Senhora da Conceição Intensive Care Unit Porto Alegre RS Brazil Intensive Care Unit, Hospital Nossa Senhora da Conceição, Grupo Hospital Conceição - Porto Alegre (RS), Brazil.; 2 Instituto D’Or de Ensino e Pesquisa Rio de Janeiro RJ Brazil Instituto D’Or de Ensino e Pesquisa - Rio de Janeiro (RJ), Brazil.; 3 Universidade Federal do Rio de Janeiro Department of Internal Medicine Rio de Janeiro RJ Brazil Department of Internal Medicine, Universidade Federal do Rio de Janeiro - Rio de Janeiro (RJ), Brazil.

## CORRELATION BETWEEN *DELIRIUM* AND SLEEP DISORDERS

Patients in intensive care units (ICUs) frequently face challenges related to *delirium* and sleep disturbances.^(1)^ Despite extensive research in recent years, *delirium* remains a complex condition with uncertain pathophysiology, and its occurrence is associated with worse outcomes as well as longer durations cognitive and functional impairment.^(1^^,^^2)^ Although no study has shown a strong relationship between ICU *delirium* and sleep to date, the development of *delirium* and sleep disturbance in the ICU is often multifactorial, with numerous related risk factors, including age, comorbidities, disease severity, environmental factors, and iatrogenic interventions.^(3)^

The lack of evidence supporting the use of pharmacological interventions (such as antipsychotics or sedatives) for *delirium* prevention or treatment in the ICU^(4)^ highlights the importance of targeted interventions to mitigate the risk of *delirium* and its predisposing conditions.^(3^^,^^4)^ Current recommendations for *delirium* prevention emphasize nonpharmacological measures, such as optimizing human care (eCASH),^(5)^ the well-established A to F bundle,^(6)^ and efforts to minimize modifiable risk factors. The PADIS guidelines maintain that sleep should be routinely monitored, and strategies for sleep hygiene enhancement should be discussed with patients.^(7)^ Despite these efforts, sleep disturbances, such as sleep deprivation, are still reported by 66% of ICU patients^(8)^ and are linked to neurocognitive dysfunction, which further increases the risk of *delirium*.^(9)^

## SLEEP QUALITY IN THE INTENSIVE CARE UNIT

Sleep in the ICU has been shown to be characterized by subjectively poor quality, high levels of fragmented sleep, and prolonged sleep latencies. Moreover, nearly 50% of ICU sleep occurs during the daytime, thus impacting rehabilitation. Although sleep is considered crucial for patient recovery, little is known about the association of sleep with physiologic function among critically ill patients or those with clinically essential outcomes in the ICU. Research involving ICU-based sleep disturbance is challenging due to the lack of objective, practical, reliable, and scalable methods to measure sleep and the multifactorial etiologies of its disruption.^(10^^,^^11)^ Electroencephalography studies have described frequent arousal, an increase in stage 2 non-REM sleep, a reduction or absence of slow-wave stage 3 non-REM sleep, and REM sleep.^(10)^

The poor quality of sleep in the ICU can be attributed to artificial light, increased noise, a consequence of critical illness, and treatment interventions that affect the day-night cycle.^(10)^ Given the challenges of improving sleep via workflow and environment redesign, pharmacological therapies with traditional sleeping pills, such as benzodiazepines, have been largely used, thus increasing the risk of developing *delirium*. Even newer nonbenzodiazepine hypnotics, such as zolpidem or atypical antipsychotics (not approved by the Food and Drug Administration for this purpose), are associated with altered mental status and in-hospital falls and may lack efficacy even in less acutely ill patients.^(11)^

## THE ROLE OF MELATONIN IN THE INTENSIVE CARE UNIT

Melatonin, a hormone produced by the pineal gland, plays a pivotal role in regulating the sleep-wake cycle. Environmental cues, especially light exposure, influence its secretion, with peak levels typically occurring at night. In the ICU, patients are often exposed to artificial lighting and noise, disrupting their circadian rhythm and melatonin production.^(10)^ The evidence of deficient melatonin levels in critically ill patients makes it theoretically reasonable to expect more significant effects of melatonin to enhance sleep quality and consequently reduce *delirium* incidence in ICU settings.^(12)^

However, despite the promising results of melatonin in improving sleep quality^(3)^ and preventing *delirium* in non-ICU settings,^(13)^ the efficacy of melatonin or ramelteon (a melatonin agonist) in preventing *delirium* in the ICU remains a topic of debate, with conflicting findings reported in recent studies. Two recently published systematic reviews and meta-analyses showed discordant results and highlighted several methodological limitations, such as the relatively low number of patients selected, heterogeneity of melatonin doses, and the use of different *delirium* assessment tools.^(3^^,^^13)^

Bandyopadhyay et al. conducted a randomized controlled trial with a 7-day follow-up to compare standard care alone or in combination with 3mg of enteral melatonin once a day. The trial was conducted in a tertiary ICU in India on patients with a clinical-surgical profile. The study involved a total of 108 patients, and measurements of the incidence of *delirium* were carried out on days 1, 3, and 7 of hospitalization in the ICU. The aim of using melatonin was to reduce episodes of *delirium* in patients. Although the study was well conducted with quality randomization and standardization of outcome assessment methods, it did not demonstrate any benefit of using melatonin to reduce the incidence of *delirium* by optimizing the sleep-wake cycle. The results of this trial add to others who did not demonstrate the benefit of using this medication as prophylaxis and/or treatment for patients with *delirium*. The author discussed *delirium* as a complex multifactorial disorder with underlying mechanisms and stated that addressing only one such mechanism (disruption of the circadian rhythm) may not be enough to determine the effect size initially aimed for in this study. However, the study did not use any method to measure the quality of sleep of patients in each group.^(14)^

## BEDSIDE STRATEGIES FOR *DELIRIUM* AND SLEEP MANAGEMENT

Nonpharmacological therapies are the cornerstone for promoting sleep quality and preventing *delirium* in the ICU. Strategies for improving sleep hygiene should be implemented in the ICU environment, including reducing noise (not exceeding 40 dB), adjusting syringe pump alarms, ensuring adequate light levels, avoiding procedures during nighttime, reviewing all current medication and the possibility of withdrawal (including nicotine or recreational addictive substances), optimizing ventilator settings, and even implementing alternative therapies for sleep promotion, including music, massage, or relaxation techniques.^(9)^ Given the multifactorial nature of these conditions, a holistic approach encompassing both pharmacological and nonpharmacological interventions is essential.

Despite the controversy regarding the use of melatonin in enhancing sleep quality and potentially reducing the incidence of *delirium*, further research is needed to clarify its efficacy and optimal dosing strategies in the ICU setting. Additionally, addressing other contributing factors beyond circadian rhythm disruption may be necessary to achieve meaningful improvements in *delirium* prevention and management.
