# Supplementary material for: Delirium and sleep quality in the intensive care unit: the role of melatonin
Source: Crit Care Sci. 2024 Apr 26;36:e20240083en. doi: 10.62675/2965-2774.20240083-en (PMC11098068; doi:10.62675/2965-2774.20240083-en)
Supplement: Translation

# Translation: *Delirium* e qualidade do sono na unidade de terapia intensiva: o papel da melatonina

**DOI:** 10.62675/2965-2774.20240083-pt

**Authors:** Pedro Henrique Rigotti Soares, Rodrigo Bernardo Serafim

**Affiliations:** 1 Porto Alegre RS Brasil Unidade de Terapia Intensiva, Hospital Nossa Senhora da Conceição, Grupo Hospital Conceição - Porto Alegre (RS), Brasil.; 2 Rio de Janeiro RJ Brasil Instituto D’Or de Ensino e Pesquisa - Rio de Janeiro (RJ), Brasil.; 3 Rio de Janeiro RJ Brasil Departamento de Clínica Médica, Universidade Federal do Rio de Janeiro - Rio de Janeiro (RJ), Brasil.

## CORRELAÇÃO ENTRE DELIRIUM E DISTÚRBIOS DO SONO

Pacientes internados em unidades de terapia intensiva (UTIs) frequentemente enfrentam dificuldades relacionadas a *delirium* e distúrbios do sono.^(1)^ Apesar de extensas pesquisas nos últimos anos, o *delirium* continua sendo uma condição complexa e com fisiopatologia incerta, e sua ocorrência está associada a desfechos desfavoráveis, bem como a uma maior duração do comprometimento cognitivo e funcional.^(1^^,^^2)^ Embora até o momento não existam estudos que demonstrem uma forte relação entre *delirium* e sono, o desenvolvimento de *delirium* e distúrbios do sono em UTIs é frequentemente multifatorial, com vários fatores de risco relacionados, incluindo idade, comorbidades, gravidade da doença, fatores ambientais e intervenções iatrogênicas.^(3)^

A escassez de evidências que apoiem o uso de intervenções farmacológicas (como antipsicóticos ou sedativos) para a prevenção ou o tratamento do *delirium* na UTI^(4)^ ressalta a importância de intervenções direcionadas para mitigar o risco de *delirium* e suas condições predisponentes.^(3^^,^^4)^ As recomendações atuais para a prevenção do *delirium* enfatizam medidas não farmacológicas, como a otimização do cuidado humano (eCASH),^(5)^ o bem estabelecido pacote A a F^(6)^ e esforços para minimizar os fatores de risco modificáveis. As diretrizes PADIS sustentam que o sono deve ser monitorado rotineiramente, sendo que as estratégias para melhorar a higiene do sono devem ser discutidas com os pacientes.^(7)^ Apesar desses esforços, os distúrbios do sono, como a privação do sono, ainda são relatados por 66% dos pacientes de UTI^(8)^ e estão ligados à disfunção neurocognitiva, o que aumenta ainda mais o risco de *delirium*.^(9)^

## QUALIDADE DO SONO NA UNIDADE DE TERAPIA INTENSIVA

Demonstrou-se que o sono na UTI é caracterizado por uma qualidade subjetivamente ruim, níveis elevados de sono fragmentado e latências de sono prolongadas. Além disso, quase 50% do sono na UTI ocorre durante o dia, o que afeta a reabilitação. Embora o sono seja considerado crucial para a recuperação do paciente, pouco se sabe sobre a associação do sono com a função fisiológica entre pacientes graves ou aqueles com desfechos clinicamente essenciais na UTI. Estudos envolvendo distúrbios do sono em UTIs são desafiadores devido à falta de métodos objetivos, práticos, confiáveis e escalonáveis para medir o sono e as etiologias multifatoriais de sua interrupção.^(10^^,^^11)^ Estudos de eletroencefalografia descreveram despertares frequentes, aumento do estágio 2 do sono não REM, redução ou ausência do estágio 3 do sono não REM de ondas lentas e do sono REM.^(10)^

A má qualidade do sono na UTI pode ser atribuída à luz artificial, ao aumento de ruído, ser uma consequência da doença crítica e se dar pelas intervenções de tratamento que afetam o ciclo dia-noite.^(10)^ Considerando os desafios de melhorar o sono por meio do redefinição do fluxo de trabalho e do ambiente, têm-se utilizado amplamente terapias farmacológicas com soníferos tradicionais, como os benzodiazepínicos, aumentando o risco de desenvolvimento de *delirium*. Até mesmo os hipnóticos não benzodiazepínicos mais recentes, como zolpidem ou antipsicóticos atípicos (não aprovados pela *Food and Drug Administration* [FDA] para essa finalidade), estão associados a estado mental alterado e quedas hospitalares e podem não ser eficazes mesmo em pacientes menos agudamente graves.^(11)^

## O PAPEL DA MELATONINA NA UNIDADE DE TERAPIA INTENSIVA

A melatonina, um hormônio produzido pela glândula pineal, desempenha papel fundamental na regulação do ciclo sono-vigília. Sinais ambientais, especialmente a exposição à luz, influenciam em sua secreção, com os níveis máximos ocorrendo normalmente à noite. Na UTI, os pacientes são geralmente expostos à iluminação e a ruídos artificiais, o que perturba seu ritmo circadiano e a produção de melatonina.^(10)^ A evidência de níveis deficientes de melatonina em pacientes graves torna teoricamente razoável esperar efeitos mais significativos da melatonina para melhorar a qualidade do sono e, consequentemente, reduzir a incidência de *delirium* em ambientes de UTI.^(12)^

No entanto, apesar dos resultados promissores da melatonina na melhora da qualidade do sono^(3)^ e na prevenção do *delirium* em ambientes que não sejam de UTI,^(13)^ a eficácia da melatonina ou da ramelteona (um agonista da melatonina) na prevenção do *delirium* na UTI continua sendo objeto de debate, com achados contraditórios relatados em estudos recentes. Duas revisões sistemáticas e metanálises publicadas recentemente mostraram resultados divergentes e destacaram várias limitações metodológicas, como o número relativamente baixo de pacientes selecionados, a heterogeneidade das doses de melatonina e o uso de diferentes ferramentas de avaliação do *delirium*.^(3^^,^^13)^

Bandyopadhyay et al. realizaram um estudo controlado e randomizado com um seguimento de 7 dias para comparar o tratamento padrão isoladamente ou associado a 3mg de melatonina enteral uma vez ao dia. O estudo foi realizado em uma UTI terciária em pacientes com perfil clínico-cirúrgico na Índia. O estudo incluiu 108 pacientes, e as medições da incidência de *delirium* foram realizadas nos dias 1, 3 e 7 de internação na UTI. O objetivo do uso da melatonina era reduzir os episódios de *delirium* nos pacientes. Embora o estudo tenha sido bem conduzido, com randomização de qualidade e padronização dos métodos de avaliação de desfechos, não demonstrou qualquer benefício para reduzir a incidência de *delirium* ao otimizar o ciclo sono-vigília. Os resultados desse estudo se somam a outros que não demonstraram o benefício do uso dessa medicação como profilaxia e/ou tratamento de pacientes com *delirium*. O autor discutiu o *delirium* como um distúrbio multifatorial complexo com mecanismos subjacentes e afirmou que a abordagem de apenas um desses mecanismos (interrupção do ritmo circadiano) pode não ser suficiente para determinar o tamanho do efeito inicialmente almejado nesse estudo. Entretanto, o estudo não utilizou nenhum método para medir a qualidade do sono dos pacientes em cada grupo.^(14)^

## ESTRATÉGIAS À BEIRA DO LEITO PARA MANEJO DO DELIRIUM E DO SONO

Terapias não farmacológicas são fundamentais para promover a qualidade do sono e prevenir o *delirium* na UTI. Estratégias para melhorar a higiene do sono devem ser implementadas no ambiente da UTI, incluindo reduzir ruídos (não superiores a 40 dB), ajustar alarmes de bombas de seringa, garantir níveis de luz adequados, evitar procedimentos durante a noite, revisar todos os medicamentos atuais e a possibilidade de abstinência (incluindo nicotina ou substâncias aditivas recreativas), otimizar as configurações do ventilador e, até mesmo, implementar terapias alternativas para a promoção do sono, incluindo música, massagem ou técnicas de relaxamento.^(9)^ Dada a natureza multifatorial dessas condições, é essencial uma abordagem holística que englobe intervenções farmacológicas e não farmacológicas.

Apesar da controvérsia quanto ao uso da melatonina para melhorar a qualidade do sono e, possivelmente, reduzir a incidência de *delirium*, é necessário realizar mais pesquisas para esclarecer sua eficácia e estratégias de dosagem ideais no ambiente da UTI. Além disso, pode ser necessário abordar outros fatores contribuintes além da perturbação do ritmo circadiano para obter melhorias significativas na prevenção e no controle do *delirium*.
